# A valve powered by earthworm muscle with both electrical and 100% chemical control

**DOI:** 10.1038/s41598-019-44116-3

**Published:** 2019-07-08

**Authors:** Yo Tanaka, Shun-ichi Funano, Yuji Noguchi, Yaxiaer Yalikun, Norihiro Kamamichi

**Affiliations:** 1 Center for Biosystems Dynamics Research (BDR), RIKEN, 1-3 Yamadaoka, Suita Osaka, 565-0871 Japan; 2 Department of Robotics and Mechatronics, Tokyo Denki University, 5 Senju-asahi-cho, Adachi-ku, Tokyo 120-8551 Japan

**Keywords:** Fluidics, Mechanical engineering, Bioenergetics

## Abstract

Development of bio-microactuators combining microdevices and cellular mechanical functions has been an active research field owing to their desirable properties including high mechanical integrity and biocompatibility. Although various types of devices were reported, the use of as-is natural muscle tissue should be more effective. An earthworm muscle-driven valve has been created. Long-time (more than 2 min) and repeatable displacement was observed by chemical (acetylcholine) stimulation. The generated force of the muscle (1 cm × 3 cm) was 1.57 mN on average for 2 min by the acetylcholine solution (100 mM) stimulation. We demonstrated an on-chip valve that stopped the constant pressure flow by the muscle contraction. For electrical control, short pulse stimulation was used for the continuous and repeatable muscle contraction. The response time was 3 s, and the pressure resistance was 3.0 kPa. Chemical stimulation was then used for continuous muscle contraction. The response time was 42 s, and the pressure resistance was 1.5 kPa. The ON (closed) state was kept for at least 2 min. An on-chip valve was demonstrated that stopped the constant pressure flow by the muscle contraction. This is the first demonstration of the muscle-based valve that is 100% chemically actuated and controlled.

## Introduction

Fusion of microelectromechanical systems (MEMS) technology and living materials (e.g. biomolecules, cells or tissue) has produced various novel devices exploiting the capability for MEMS to accommodate different sizes and processing capabilities. While most of them are chemical or biochemical analysis devices which are often referred to as cells-on-chips^1,2^ or organ-on-chips (human-on-chips)^3–5^, recent studies have also exploited the cellular mechanical functions to construct bio-microactuators with living materials: that is, naturally integrated, biocompatible and efficient driving components driven by chemical energy (ATP)^6–10^. For example, microorganisms such as bacteria or protozoa have been used to create ultra-small-scale actuators^11–13^. For more dynamic actuation, living muscle tissue has been utilized. A number of cardiomyocyte robots^14–17^ and skeletal muscle cell robots^18–20^ have been reported. However, most of these devices use cultured cells. In case of using cultured muscle cells, a substrate membrane is usually required. The contractile force of the muscle cells is also used to strain the membranous sheet. Therefore, compared with natural muscle tissue, the contractile force becomes weak.

On the other hand, we have previously utilized as-is natural muscle tissue, namely muscle tissue of earthworms, and we demonstrated an on-chip micropump that employed it^21^. Although other studies have also realized micropumps by fabricating membranous actuators using cell sheets^22–24^ or using thin polydimethylsiloxane (PDMS) membrane as scaffolds^25,26^, they use cultured cells which have weaker force. Moreover, the actuating motion of cardiomyocytes is difficult to control because they beat spontaneously. By contrast, natural earthworm muscle can generate a large force, and also has a short response time and controllability like skeletal muscle tissue.

However, a valve has never been realized which is one of the indispensable components for fluid control systems as well as pumps. To realize a valve, it is indispensable to produce large force and displacement in addition to the continuous contractile motion and ON-OFF switching availability with arbitrary timing. Furthermore, conventional bio-microactuators including the earthworm muscle-based pump are ON-OFF controlled by electricity or light. This fact means that they were not independent of an external electricity supply; such independence is one of the most significant properties of living materials. To address these issues and to keep the contractile state, chemical stimulation is suitable. Although the momentary maximum contractile force of tissue is weaker than that with electrical stimulation, long-term (over 1 min) duration can be achieved as seen in the chemical stimulation response of smooth muscle cells^27^ or electrocytes^28^.

Based on this concept, our aim in this report was to verify the working principle of an earthworm muscle-based valve on a microfluidic chip. First, the mechanical motion of the earthworm responding to the chemical stimulation was investigated. Second, the valve function using short pulse electrical stimulation was demonstrated to prove the potential of the earthworm muscle to be used for a valve. Third, the valve function using chemical (acetylcholine) stimulation was demonstrated.

**Figure 1:**
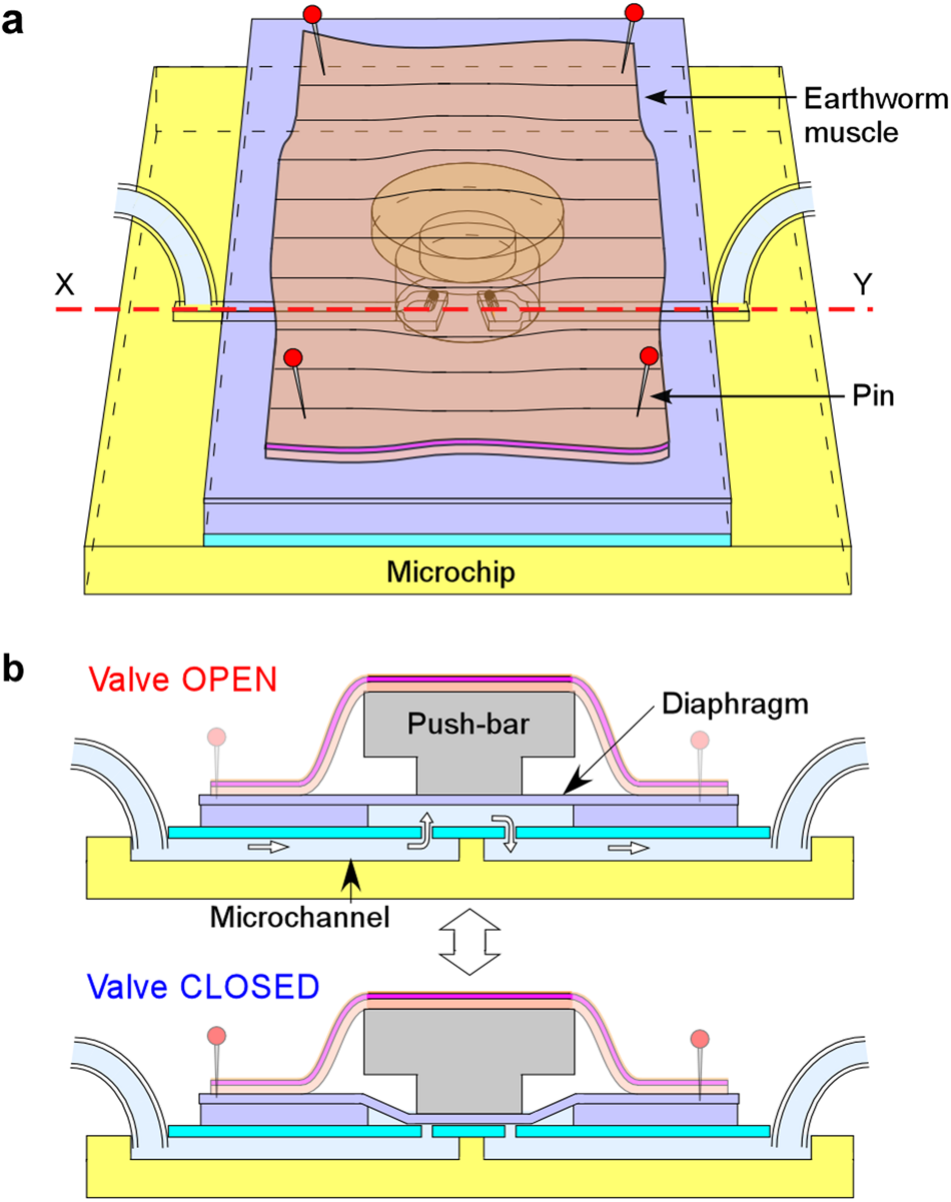
A valve on a chip powered by earthworm muscle. (**a**) Design and working principle of the valve as a schematic view. (**b**) A cross-sectional schematic view along line X-Y in (**a**) when the valve is open (upper) and closed (bottom).

Figure 1 shows the design and the driving principle of a normally open type valve on-chip with an on-board earthworm muscle sheet working as a fluid driving actuator. The fundamental design was similar to that of our previously reported earthworm-driven pump^21^, but it was customized for a valve system. The muscle, cut to a rectangle of about 2 cm × 1 cm, was fixed onto a microchip using pins at each of the corners. A thin diaphragm and a push-bar structure were installed on the microchip to transmit the continuous, contractile force of the muscle sheet to the fluid in the microchip. When the valve is OFF, fluid is introduced with constant pressure from the inlet using a compressor via a pressure regulator. When the earthworm muscle is stimulated electrically or chemically, it is contracted and the two penetrating holes connecting the microchannel and the valve chamber are closed by the diaphragm via the push-bar structure, and then the valve becomes ON (closed). All of the microchip components are made of PDMS, and the microchip size is 2 cm × 2 cm square.

## Results

### Measurement of contraction force by chemical stimulation

**Figure 2:**
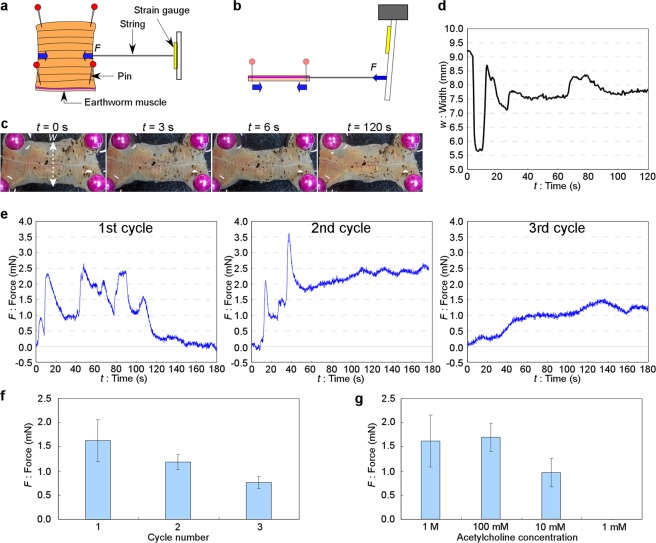
Measurement of the contractile force of the earthworm muscle by chemical stimulation. (**a**) A schematic illustration showing the measurement system. A string was directly connected to the muscle sheet, and the lateral contractile force was measured by the motion of a cantilever on which a strain gauge was attached. (**b**) A cross-sectional view along the horizontal center line in (**a**). (**c**) Observation of muscle contraction before (*t* = 0 s) and after (*t* = 3, 6, and 120 s) stimulation by 100 mM acetylcholine application. (**d**) Time-course of the width of the center-line of the muscle (*w*) in (**c**) for 2 min. (**e**) Time-courses for the contractile force for 3 min after stimulation. Acetylcholine solution (100 mM) was applied at about *t* = 0 s. The contractile force at *t* = 0 s was defined as 0 mN. Results of the first, second and third cycles with the same sample after washing with PBS solution each time are displayed. (**f**) Contractile force versus cycle number of repeat trials with the same sample at the condition of 100 mM acetylcholine stimulation (n = 3 ± s.e.m.). (**g**) Contractile force (average during 2 min after stimulation) of the earthworm muscle versus acetylcholine concentration (n = 3 ± s.e.m.).

We investigated the fundamental contractile motion behavior and the repeatability of the earthworm muscle responding to chemical stimulation using the contractile force measurement system (Fig. 2a,b). We used a well-known neurotransmitter: acetylcholine which acts on the muscle to contract. The muscle sheet (1 cm × 3 cm) was fixed by pins in 4 corners. In this state, the middle part of the muscle was free to move.

First, to observe the response of the muscle, we observed how much the muscle shrank after adding acetylcholine solution without attaching a string to the muscle. Phosphate buffered saline (PBS) solution was used as a solvent. As shown in Fig. 2c and in Supplementary Movie 1, significant shrinkage (decrement of the width) of the muscle was observed within a few seconds after applying acetylcholine solution (100 mM). The width of the center-line of the muscle (*w*) indicated in Fig. 2c was measured and the time-course every 0.5 s was plotted in Fig. 2d. The widths of the muscle from the movie images were measured using straight tool of ImageJ software. Although the shrinkage length of the earthworm muscle was fluctuated and not stable, it was roughly recovered to the initial length after 2 min. This result means that enough displacement was obtained for the muscle to be used for actuators by chemical stimulation.

Then, to quantitatively measure the direct pulling force of the muscle, we utilized the force measurement system that had a cantilever-type force transducer (strain gauge). A string was connected to the middle part of the earthworm muscle, and the contractile force was passed to the cantilever (2-cm width and 1-mm thickness) via the string., The strain gauge was on the cantilever, 10 cm from the cantilever root. By measuring the resistance of the strain gauge, the contractile force was calculated.

We measured the time-course of the contractile force of the earthworm muscle for 3 min after applying acetylcholine solution (100 mM) to cover the muscle. After that, the muscle was completely washed with a sufficient amount of PBS solution to remove the remaining acetylcholine and that was followed by applying the acetylcholine solution stimulation again to confirm the recovery of the muscle. The experiment using the same sample was carried out 3 times (3 cycles).

As shown in Fig. 2e, the muscle responded within a few seconds after the application of acetylcholine solution. Even though the contractile force fluctuated, probably because of the spontaneous, random actuation of earthworm muscle, a force of at least 1 mN continued to be generated for about 2 min, and the time-averaged force during 2 min was 1.49 mN. After washing with PBS and making the second application of acetylcholine solution (second cycle), continuous contractile motion was observed again for more than 2 min. In the third cycle, the contractile force was less and it took a longer time to reach 1 mN; however, some contractile force was observed. Therefore, the continuous contractile motion was observed for at least 3 times.

The response of the muscle to chemical stimulation in each cycle had much variation in behavior because it was living. Natural, random motion of the muscle remained in relatively long-time scale in case of using chemical stimulation compared with electrical one^21^. This caused fluctuation of the measured force (especially as seen in Fig. 2d and in the 1st cycle of Fig. 2e). Therefore, the measured force was not similar in each cycle. The duration of the contractile state was also fluctuated and difficult to be measured by the same reason. Roughly, the force was gradually decreased and almost returned to the original state within 5 minutes in our data.

In this experiment, PBS (without acetylcholine) was used for relaxation of the muscle. Although the relaxation profile should also be measured, it was difficult in the current system. This is because through washing at least 3 times was necessary to remove acetylcholine enough and to recover the muscle to be the original relaxing state differently from just adding the acetylcholine solution when stimulating the muscle. This operation frequently changed the position the muscle. Therefore, the same initial tension was difficult to be maintained without influenced by this disturbance. Furthermore, the muscle did not always return to the same state even after thorough washing. Here, just the repeatable usability of the muscle by washing with PBS was confirmed and relative contractile force in each cycle was measured. In each cycle, the force at *t* = 0 s (just before applying acetylcholine) was defined as *F* = 0 N.

To compare the contractile force versus the cycle number, we plot the time-average values of the force during 2 min in each cycle in Fig. 2f. The force (average of 3 samples) gradually decreased, but repetitive contraction was confirmed. The effect of the acetylcholine concentration is shown in Fig. 2g. For 10 mM acetylcholine application, contractile force was detected. For 1 mM acetylcholine application, almost no effect was observed. On the other hand, the highest concentration was 1 M due to the limit of acetylcholine solubility. The force at 1 M was almost not changed (or rather decreased) compared with 100 mM. The stability and duration were also not significantly changed. Considering the complicated operations using higher concentration to make the muscle relax, it was not changed to use 100 mM concentration for the following experiments.

In this experiment, we found that the earthworm muscle generated a large contractile force (more than 1 mN) and the continuous, repetitive actuation was possible with chemical stimulation. The average force of 3 samples was 1.57 ± 0.26 mN (n = 3, error indicates s.e.m.). Compared with the momentary maximum force in response to an electrical stimulation for the same condition (about 7.82 mN)^21^, the value of the force was smaller, but was still within the same order. From these results, we concluded that chemical stimulation could be used to drive a valve.

### Valve demonstration by electrical stimulation

Before creating a chemically controlled valve, we demonstrated the function of a valve operated by electrical stimulation to verify that earthworms could be used for valve actuation, because electricity has higher controllability and produces a larger contractile force as described above.

We used consecutive pulse type stimulation for the valve demonstration. This is because the force of the muscle gradually decreased in case of applying constant electricity (6 V), while consecutive pulse type stimulation kept the contraction steady without decrement as shown in the previous study.^21^ The contractile pulling motion of the muscle was transduced into the pushing motion using a push-bar structure, and this pushing force was measured (Supplementary Fig. S1). This structure is the same as that for the valve system shown in Fig. 1b. While the force test in Fig. 2 measured the lateral force of the muscle to know the maximum performance of natural muscle behavior as a fundamental investigation for biological property, that is a pulling force, not a pushing force. To make a valve, a pushing force is indispensable to surely close holes in a valve chamber. The result indicated that the contractile force of about 0.80 ± 0.03 mN (n = 3, error indicates s.e.m.) was sustained while the consecutive pulse signal (0.1 s duration and 0.2 s interval) was applied for 10 s. This repetitive contracting-relaxing state transition is suitable for valve actuation^21^. The pushing force was about 1 order smaller than the lateral pulling force because the force was communicated not directly but via the push bar structure.

To determine whether the measured force (0.80 mN) is usable for valve operation, we analyzed how much fluid pressure could be stopped by this force. The force applied to the push-bar by the fluid from the penetrated, inlet hole connecting the microchannel and valve chamber (*F*) was calculated by the equation: *F* = *P* (π*r*^2^), where *P* is the fluid pressure and *r* is the radius of the penetrated hole connecting a microchannel and a chamber. To balance the force of the push-bar and fluid (*F* = 0.80 mN), *P* is calculated to be 25 kPa using the design parameter, *r* = 100 μm. This result means that the theoretical pressure resistance is 25 kPa, which is enough to demonstrate the valve function as described in the Discussion section in detail.

**Figure 3:**
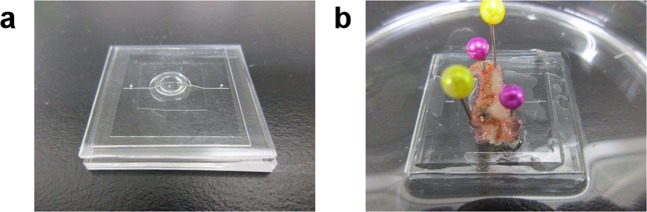
Fabricated microchip for valve demonstration. (**a**) Photo of the fabricated PDMS microchip for the earthworm valve demonstration. (**b**) Photo of the microchip after positioning the earthworm muscle on it.

Based on this consideration, we constructed an actual on-chip valve. A PDMS microchip (3-mm chamber diameter) was fabricated as described in the Methods section. The fabricated microchip and the earthworm muscle sheet were assembled as shown in Fig. 3a,b, respectively. The size of the muscle sheet was 2 cm × 1 cm to be fitted to the microchip size. The DC electricity was supplied via the pins used for fixing the muscle in place; the pins also worked as electrodes. The applied voltage was pulse type (0.1 s duration and 0.2 s interval). This short-pulse voltage application time was 30 s followed by 10 s rest for valve actuation. Applied voltage was 6 V.

**Figure 4:**
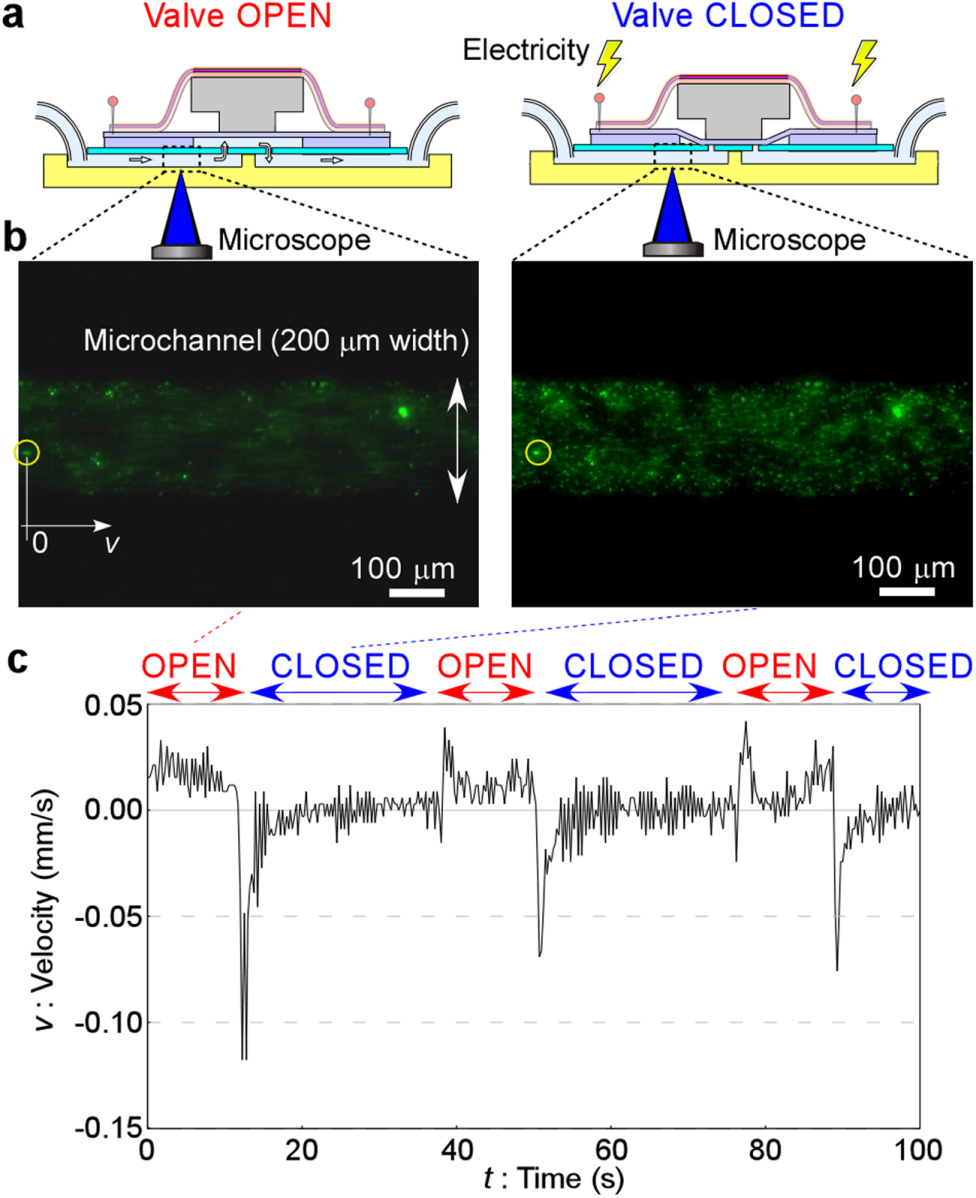
Demonstration of an earthworm muscle-based electrically controlled valve. (**a**) Cross section showing the method to measure flow speed in a microchannel and the states when the valve is open and closed. (**b**) Fluorescence microscope observation of fluid in a microchannel visualized with fluorescently labeled microparticles (green points) showing valve open (flowing) and closed (stopped) states. (**c**) Velocity time-courses during 100 s for the selected particle (indicated by a circle in **b**) when short pulse type voltage was applied. Valve open and closed states are indicated across the top of the graph.

As shown in Fig. 4a, fluid motion was monitored by following the fluorescent polystyrene tracking particles to demonstrate stopping and re-flowing of fluid by the valve (Supplementary Movie 2). Observations were made at the microchannel located before reaching the valve chamber (inlet channel) on the chip. Fluid was introduced into the microchannel at 0.1 kPa, which was the minimum pressure of the pressure controller to trace the particle movement as precisely as possible. The displacement time-course trajectory directly observed by the video recording for a selected particle (the particle marked by a circle in Fig. 4b) is plotted by the line in Fig. 4c. Particle velocity to the right (*v*) was measured directly from sequential frames of the microscope video image every 0.25 s.

In Fig. 4c, constant motion of the particle was observed before *t* = 10 s, but the net movement stopped after a quick back flow caused by compression of the valve chamber during the closing motion of the stop valve around *t* = 10 s. Even though some fluctuation was observed due to the short pulse type voltage application, the particle almost stopped (valve closed) during *t* = 10–40 s corresponding to the voltage application time. During *t* = 40–50 s corresponding to the non-voltage application time, re-flowing of the particle in the normal flow direction was observed, which means that the valve re-opened. This cycle was repeated for 3 times, and almost the same motion was observed each time. The average response time from the data of these 3 cycles was about 3 ± 0 s (n = 3, error indicates s.e.m.). Furthermore, when the applied pressure was increased in 0.1 kPa steps, the valve worked well without leakage up to 3.0 kPa which indicates the pressure resistance of this valve. Even though this value is smaller than the theoretical pressure resistance calculated above, probably because of a non-uniform force distribution and the presence of material structures, they are not so far away.

Overall, these results clearly demonstrate the electrically controlled and repeatable stop valve function using the earthworm muscle sheet.

### Valve demonstration by chemical stimulation

Following the fundamental investigations described above, we next demonstrated chemical control of an on-chip earthworm valve using acetylcholine. The prepared PDMS microchip was similar to that used for the valve demonstration by electrical stimulation. However, the diameter of the valve chamber was enlarged from 3 to 8 mm. This was because the valve did not work using the 3-mm diameter chamber due to the larger force requirement to close the valve with a smaller chamber.

A piece of fresh earthworm muscle was positioned on the microchip. We observed fluid flow in the microchannel for 2 min after applying acetylcholine solution to cover the muscle. The experiment using the same sample was carried out for 3 cycles. In each cycle, the muscle was completely washed with a sufficient amount of PBS solution to remove the remaining acetylcholine.

**Figure 5:**
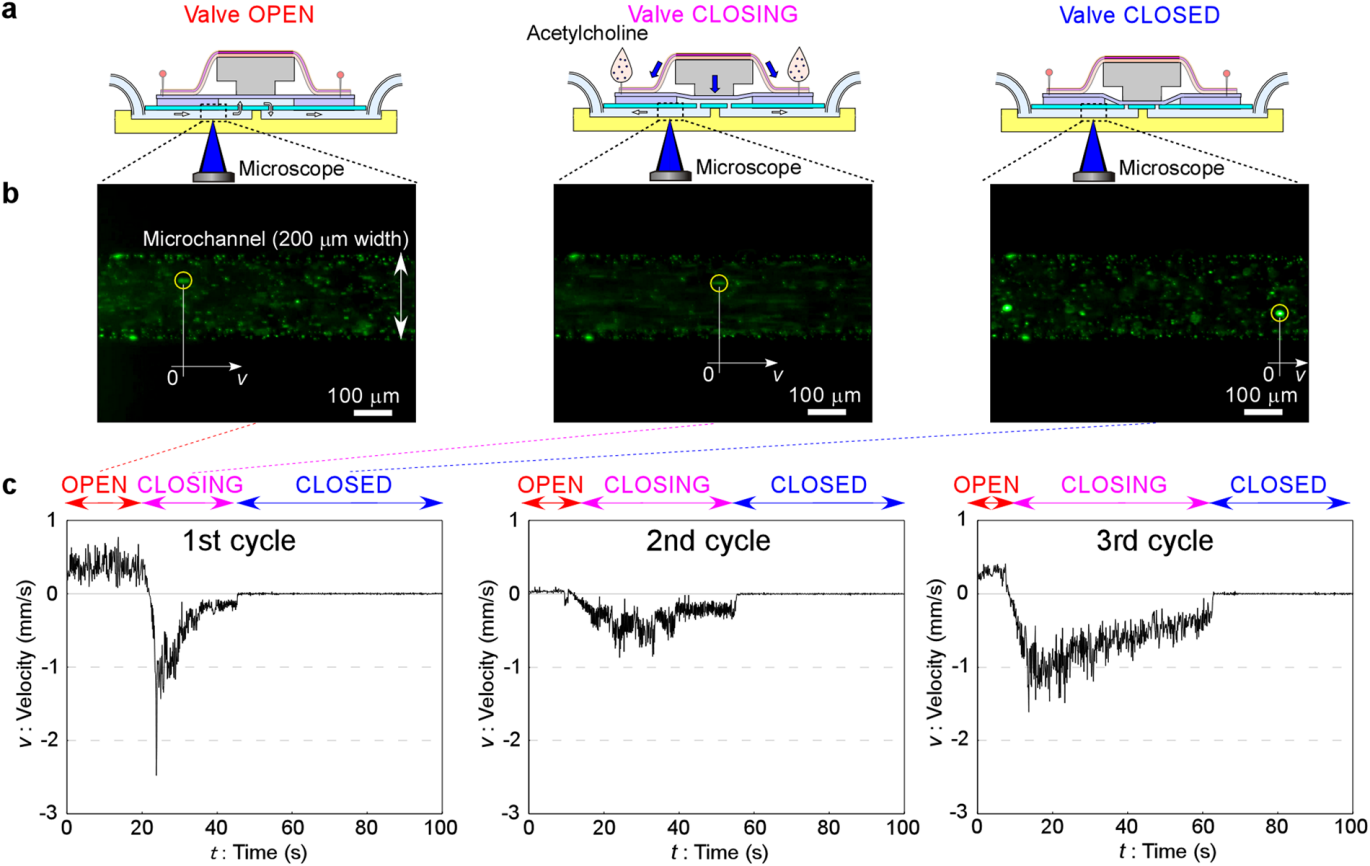
Demonstration of earthworm muscle-based chemically controlled valve. (**a**) Cross section showing the states when the valve is open, closing and closed. (**b**) Fluorescence microscope observation of fluid in a microchannel visualized with fluorescently labeled microparticles showing valve open (flowing forward), closing (flowing backward) and closed (stopped). (**c**) Velocity time-courses during 100 s of selected particles (indicated by circles in (**b**). The first, second and third cycles with the same sample after washing with PBS solution each time are presented, and acetylcholine solution was applied at about *t* = 20, 8 and 7 s, respectively. Valve open, closing and closed states are indicated across the top of the graph.

Fluid motion was monitored by following the particles the same as in the case of the electrical valve (Fig. 5a), and fluid was introduced into the microchannel at 0.5 kPa. The displacement time-course trajectories directly observed by the video recording for selected particles (particles marked by circles in Fig. 5b) are plotted by the line in Fig. 5c. Particle velocity to the right (*v*) was measured directly from sequential frames of the captured microscope video image every 0.10 s. Differing from the electrically controlled valve, however, we found the displacements of the particles were so large that it was impossible to trace just one particle for a few minutes. Therefore, the tracking particle was changed after the originally tracked particle moved from the field of view. A particle moving at a velocity nearly equal to the previously traced particle was newly selected.

In Fig. 5c (first cycle, Supplementary Movie 3), constant motion of the particle was observed initially. This means that the valve was open (particles moved forward) before *t* = 10 s. Acetylcholine solution was applied around *t* = 10 s. During *t* = 10–45 s, the valve was closing (particles moved backward). In this context, “closing” means the state “on the way from open to closed”. This closing time was longer than that for the valve controlled by electrical stimulation. This is probably because of slowness of the earthworm muscle to respond to the chemical stimulation in addition to the use of a larger valve chamber (8 mm in diameter) of the microchip compared with the electrically controlled valve (3 mm in diameter). After *t* = 45 s, the valve was completely closed (particles stopped moving). This valve closed state was kept for at least 2 min every time. To decrease the back-flow, minimizing the chamber diameter and depth is one of the possible methods.

In the second (Supplementary Movie 4) and third (Supplementary Movie 5) cycles, similar motions were observed. The valve was not closed initially in the 2nd cycle, although the speed of the particles was slow. Recordings of the fluid motion were stopped once between the cycles because it was difficult to wash the muscle with PBS without moving the setup, but re-flow at the initial moment (*t* = 0 s) in the second and third cycles means that the valve re-opened each time. The difference of the initial flow speed in each cycle might be because of the difference of the initial tension of the muscle after washing acetylcholine in each cycle as also described in “Measurement of contraction force by chemical stimulation” section. However, this phenomenon did not affect the valve performance confirmation. This cycle was repeated 3 times, and similar motion was observed each time. The average response time indicated by these data was about 42 ± 9 s (n = 3, error indicates s.e.m.).

When the applied pressure was increased in 0.5 kPa steps, the valve worked well without leakage up to 1.5 kPa which indicated the pressure resistance of this valve. This value is smaller than the pressure resistance of the electrically controlled valve (3.0 kPa) because of the difference in the force by generated electrical and chemical stimuli as described in “Measurement of contraction force by chemical stimulation” section. The difference of the chamber diameter would also affect the pressure resistance. In other words, the valve controlled by electrical stimulation is potentially higher than the valve controlled by chemical stimulation if the valve chamber with the same diameter was used.

These results clearly demonstrate the chemically controlled and repeatable stop valve function using the earthworm muscle sheet.

## Discussion

Here, we compare the performance of our valve and that of previously reported valves. Several types of valves have been reported for microfluidic systems. Table 1 summarizes them in a comparison of control methods, drivers, pressure resistance and response time.

**Table 1:** Performance comparison of the developed valve with other actively controlled valves regarding energy source, driver, pressure resistance, and response time (“n/r” denotes “not reported”).

Control method	Reference	Driver	Pressure resistance (kPa)	Response time (s)
Elctrical	This study	Earthworm	3.0	3
	29	Piezo (Bimorph)	3.0	0.1
	30	Electroactive polymer	4.0	0.7
Chemical	This study	Earthworm	1.5	42
	31	Hydrogel (pH)	n/r	8
	32	Hydrogel (pH)	39	n/r
Pnumatic	33	Air	60	0.01
Optical	34	Poly(N-isopropylamide)	n/r	5
	35	Poly(N-isopropylamide)	10	30
Magnetic	36	Permanent magnet	200	0.05
	37	PDMS-iron	n/r	n/r
Thermal	38	PDMS-silver	200	23
	39	Polyurethane	200	10
	40	Polyethylene glycol	10	60
	41	Thermo responsive fluid	10	7–80

A number of on-chip active valves (driven and controlled via external device operation) that exploit the volume change in microchannels or valve chambers have been developed, including electrically^29,30^, chemically^31,32^, pneumatically^33^^, optically^^34,35^^,^ magnetically^36,37^, and thermally^38–41^ actuated (switched) valves. Valves with smaller size channels have shorter response time and larger pressure resistance^42,43^, but only valves with relatively large displacement (~100 μm) accompanied with volume change were included in this comparison.

Generally, electrically, pneumatically, and magnetically controlled valves have short response time (within 1 s). Compared with these valves, the earthworm muscle-driven valve moves slowly. On the other hand, chemically, optically and thermally controlled valves have relatively low response time due to slow volume change speed of a driving polymer or hydrogel or low sensitivity of these valves in responding to external stimuli, even though some of them have high pressure resistance. Compared with chemically, optically and thermally controlled valves, the earthworm muscle-driven valve (even though with chemical control) is not so slow. Regarding the pressure resistance, it differs significantly depending on the channel geometry or the materials rather than physical or chemical properties of the driver itself. Overall, the pressure resistance and the response time are considered to be included in the range of reported valves used for microfluidic experiments, and therefore the earthworm muscle-driven valve has a potential to be used for microfluidic systems.

From the viewpoint of driving energy for switching the valve, most of the valves except for chemical stimuli-based valves originally depended on electricity. Moreover, the chemically stimulated valves^31,32^ were controlled by pH change (acid). Therefore, it is difficult to use them for bio-related applications like other electricity-dependent valves. Differing from these valves, the earthworm muscle valve is driven by ATP and controlled by acetylcholine, both of which are quite common materials in a living body environment. So, the earthworm muscle valve driven by ATP can be considered as a bio-friendly device.

We designed and demonstrated an earthworm muscle-driven valve controlled both electrically and chemically. First, we investigated the fundamental contractile force of an earthworm muscle sheet responding to the chemical stimulation using the neurotransmitter, acetylcholine. Long-time (more than 2 min) and repeatable displacement was observed. The generated force of the muscle (1 cm × 3 cm) was 1.57 mN in average for 2 min for stimulation by acetylcholine solution (100 mM). Second, an on-chip valve that used the earthworm muscle sheet was demonstrated to be able to stop the constant pressure flow by the muscle contraction. For electrical control, short pulse stimulation was used for the continuous muscle contraction. The response time was 3 s, and the pressure resistance was 3.0 kPa. For chemical control, the valve function using acetylcholine stimulation was demonstrated. The response time was 42 s, and the pressure resistance was 1.5 kPa. The ON (closed) state was kept for at least 2 min. In both electrically and chemically controlled methods, repeatable valve function was also demonstrated.

This is the first demonstration of the cell-based valve, and also the 100% chemically actuated and controlled bio-microactuator. In a living body, vascular systems control blood flow by using the continuous contraction of smooth muscle cells surrounding the blood vessels responding to the released chemical molecules from inside endothelial cells without any external control. Likewise, this device can be applied to a living, autonomous fluidic control system in the future. To realize such applications, the use of cultured cells should be also considered to improve the reproducibility or to enable mass production. Moreover, cultured-cell based bio-devices are easily designed with various optimum shapes for applications by fabricating composites of cultured cells and artificial materials. For practical use, constructing cultured tissue like earthworm muscle will be required.

## Methods

### Preparation of earthworm muscle

As in a previous report^21^, earthworms of *Lumbricus terrestris* (a large, reddish worm species) were used. Briefly, an earthworm was cooled for about 15 min on ice to anesthetize it. The earthworm was placed on a spongy board and both ends of the earthworm were stabbed with pins in order to anchor it in place. Then, the earthworm was cut using scissors in the radial direction to get a 1-cm high ring. After that, the ring was cut on the back-side area of the earthworm in the vertical direction in order to make a sheet.

### Contractile force measurement system

To measure the contractile force, a strain measurement system (NR-ST04 and NR-600, Keyence, Osaka, Japan) and interfaced software (WAVE LOGGER PRO, Keyence) were used. The time resolution was set as 1 ms. The strain was measured by using a strain gauge (KFW-5-120-C1-11, Kyowa, Chofu, Japan). All experiments were carried out at room temperature.

### Microchip fabrication

The PDMS microchip consisted of five components: a microchannel layer, a vertical, penetrated hole layer, a valve chamber layer, a diaphragm, and a push-bar. The microchannel layer was fabricated using the replica molding method and a silicon wafer^44^. PDMS prepolymer (Silpot 184 W/C, Dow Corning Toray, Tokyo, Japan) and photoresist (SU-8 3050, Nihon Kayaku, Tokyo, Japan) were used for this procedure. The depth and width of the microchannel were approximately 200 µm, and 200 µm, respectively.

The vertical, penetrated hole layer was fabricated by making holes (200 µm diameter) in a 1-mm thick PDMS sheet by using a drill. The chamber layer was fabricated by cutting a designed diameter circle (3 mm for an electrically controlled valve, 8 mm for a chemically controlled valve) in the 500-µm thick PDMS sheet. The push-bar for assembly on the 100-µm thick diaphragm was fabricated by using a biopsy punch to cut a designed diameter circle cylinders (2- and 4-mm in diameter for an electrically controlled valve, 4- and 6-mm in diameter for a chemically controlled valve) from the 500-µm thick PDMS sheet and stacking the 2 cylinders.

The PDMS microchip was assembled by the following procedure. A vertical, penetrated hole layer, a valve chamber layer, and a diaphragm were treated with vacuum oxygen plasma at an intensity of 10 W and oxygen flow rate of 8 mL/min for 30 s in the chamber of a compact etcher (FA-1, SAMCO, Kyoto, Japan). The components of the PDMS microchip were stacked in a vertical alignment and heated at 96 °C for 2 h in order to bond the components to each other. Two holes (inlet and outlet) of 500 µm in diameter were made in the edge of the microchip (microchannel) by using a biopsy punch in order to introduce liquid into the microchannel layer via a capillary tube. The components and the microchannel layer were treated with vacuum oxygen plasma at an intensity of 10 W and oxygen flow rate of 8 mL/min for 30 s in the chamber of the compact etcher and stacked in a vertical alignment and heated at 96 °C for 2 h.

### Electrical stimulation method

To stimulate the muscle electrically, a DC stabilized power supply system (AD-8723D, A&D Company, Tokyo, Japan) was used. To measure and record the applied voltage and force, a data logger was used (NR-600, Keyence) with a high-speed analogue measurement system (NR-HA08, Keyence).

### Chemical stimulation method

For chemical stimulation, acetylcholine chloride (011-00592, FUJIFILM Wako Pure Chemical Corporation, Osaka, Japan) was used. It was dissolved in Dulbecco’s PBS (048-29805, FUJIFILM Wako Pure Chemical Corporation) to the desired concentration which avoids expansion of tissue due to osmotic pressure. PBS is widely used in experiments treating tissue.

### Pressure regulation and fluid flow observation

Fluid was controlled by a microfluidic flow controller (MFCS, Fluigent, Le Kremlin Bicetre, France), and the pressure was kept constant. The PDMS microchip and a liquid tank controlled by the MFCS were connected by inserting a perfluoroalkoxy alkane (PFA) tube (IWASE, Kanagawa, Japan) (tube inner diameter, 0.4 mm; outer diameter, 0.6 mm; and length, 60 cm) extending from the outlet of the liquid tank into the hole made for introducing the liquid to the microchannel layer. Micro tracking particles were used to visualize fluid flow. Fluorescent spherical polystyrene particles (Fluoro Spheres, 2 µm diameter, Molecular Probes, Thermo Fisher Scientific, Waltham, MA, USA) were dispersed into the fluid (diluted 100× in distilled water) and fluid flow was observed *in situ* using a fluorescence microscope (IX-71, Olympus, Tokyo, Japan) with an objective lens (10×, 0.30-NA) and a charge-coupled device (CCD) camera (24-bit RGB color) (DP72, Olympus, Tokyo, Japan) and filters (filter combination of excitation/emission is 460–495 nm (bandpass)/510 nm (longpass)). The microscope was focused on the center of the inlet microchannel and the image was recorded using interfaced software (cellSens, Olympus, Tokyo, Japan) through the CCD camera^.^ All experiments were carried out at room temperature.

### Image analysis

The acquired video images were analyzed by using movie editing software (PowerDirector 12, CyberLink, New Taipei City, Taiwan). Every frame was captured and pasted into drawing software (Canvas 15, ACD systems, Fort Lauderdale, FL, USA), and particles (displacement in horizontal direction) were tracked using the still images.

## Supplementary information

Supplementary Information

Movie 1

Movie 2

Movie 3

Movie 4

Movie 5
