# Supplementary material for: A valve powered by earthworm muscle with both electrical and 100% chemical control
Source: Sci Rep. 2019 Jul 8;9:8042. doi: 10.1038/s41598-019-44116-3 (PMC6614428; doi:10.1038/s41598-019-44116-3)
Supplement: Supplementary file 1 — Supplementary Information [file 41598_2019_44116_MOESM1_ESM.doc]

**Supplementary Information**

# A valve powered by earthworm muscle with both electrical and 100% chemical control

**Yo Tanaka*^1^, Shun-ichi Funano^1^, Yuji Noguchi^1,2^, Yaxiaer Yalikun^1^ & Norihiro Kamamichi^2^**

^1^ Center for Biosystems Dynamics Research (BDR), RIKEN, 1-3 Yamadaoka, Suita, Osaka 565-0871, Japan

^2^ Department of Robotics and Mechatronics, Tokyo Denki University, 5 Senju-asahi-cho, Adachi-ku, Tokyo 120-8551, Japan

*****To whom correspondence should be addressed: E-mail: yo.tanaka@riken.jp.

TEL: +81-6-6105-5132, FAX: +81-6-6105-5132

**Table of Contents:**

- **Supplementary Figure (Fig. S1) and Caption**
- **Supplementary Movies (Movies 1-5)**
- **Supplementary Movie Captions**

**Supplementary Figure and Caption**


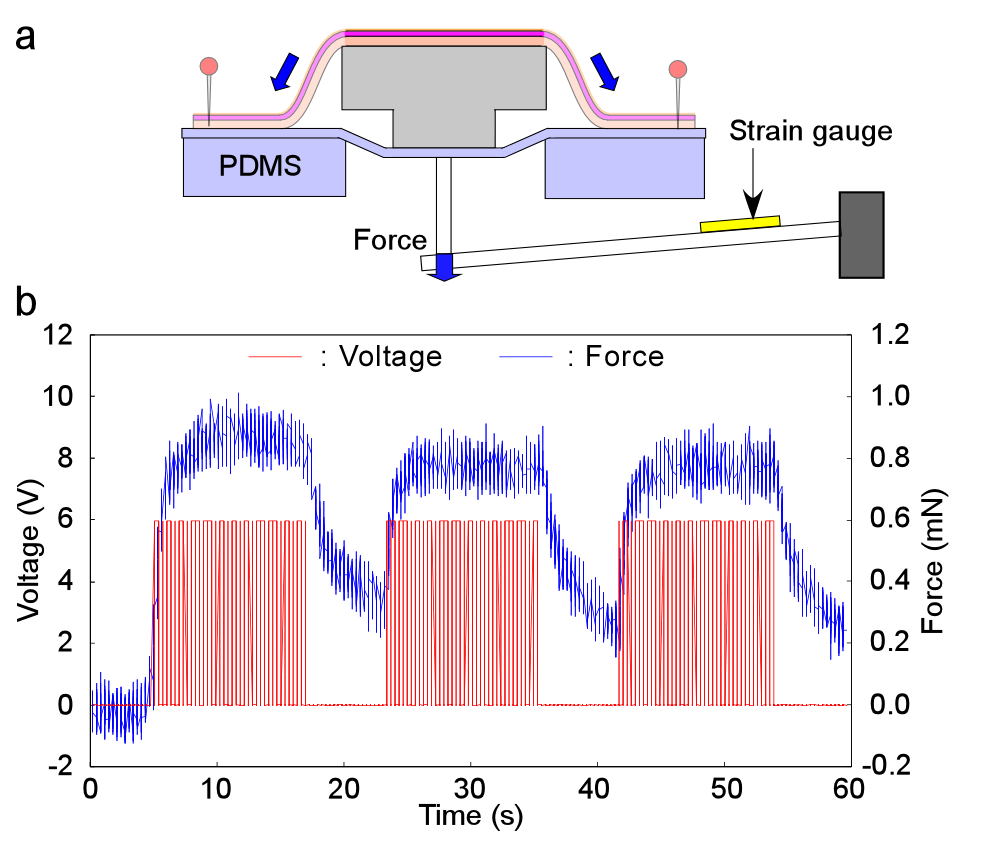


**Figure S1 |** **Measurement of the pushing force of earthworm muscle via a push-bar structure.**  (a) A cross-sectional schematic illustration showing the method using a push-bar to measure the pushing force generated by the muscle. (b) Time-course over 60 s showing the measured force. Voltage from a DC power supply was applied as a consecutive pulse stimulation of 0.1 s duration, with a 0.2 s interval between applications for 10 s followed by a 5 s rest; this represented 1 cycle. The graph is partially trimmed from Ref. 21, Figure 4.

**Supplementary Movie Captions**

**Movie 1 |** Observation of muscle contraction by 100 mM acetylcholine stimulation for 2 min. Just after starting the video recording, acetylcholine solution was applied. The initial width of the muscle was about 1 cm.

**Movie 2 |** Demonstration of an electrically controlled earthworm valve indicating real-time fluid flow in a linear microchannel (200 µm depth and width). Flow was visualized *in situ* using a fluorescence microscopy and fluorescent polystyrene tracking particles. Initially, the fluid flowed at a constant rate. Pulse type voltage was applied during *t* = 10-40 s, 50-80 s, and 90-120 s. In these time ranges, the valve was closed (particles stopped moving). In other time ranges, the valve was open (the particles were moving). Inset: Cross-sectional view of the valve showing the open and closed states.

**Movie 3 |** The first cycle in the demonstration of a chemically controlled earthworm valve indicating the real-time fluid flow in a linear microchannel. Initially, the fluid flowed at a constant rate. In this time range, the valve was open (particles were moving forward). Acetylcholine solution (100 mM) was applied around *t* = 20 s. During *t* = 20-45 s, the valve was closing (particles were moving backward). After *t* = 45 s, the valve was closed (particles stopped moving). Inset: Cross-sectional view of the valve showing the open, closing and closed states.

**Movie 4 |** The second cycle in the demonstration of a chemically controlled earthworm valve. Initially, the fluid flowed at a constant rate. Acetylcholine solution (100 mM) was applied around *t* = 8 s. During *t* = 8-55 s, the valve was closing. After *t* = 55 s, the valve was closed. Inset: Cross-sectional view of the valve showing the open, closing and closed states.

**Movie 5 |** The third cycle in the demonstration of a chemically controlled earthworm valve. Initially, the fluid flowed at a constant rate. Acetylcholine solution (100 mM) was applied around *t* = 7 s. During *t* = 7-62 s, the valve was closing. After *t* = 62 s, the valve was closed. Inset: Cross-sectional view of the valve showing the open, closing and closed states.
